# Rationale and Clinical Implications of Fluorescein-Guided Supramarginal Resection in Newly Diagnosed High-Grade Glioma

**DOI:** 10.3389/fonc.2021.666734

**Published:** 2021-05-26

**Authors:** Linda M. Wang, Matei A. Banu, Peter Canoll, Jeffrey N. Bruce

**Affiliations:** Gabriele Bartoli Brain Tumor Laboratory, Department of Neurological Surgery and Department of Pathology and Cell Biology, Columbia University Irving Medical Center, New York, NY, United States

**Keywords:** fluorescein, supramarginal resection, supramaximal resection, glioblastoma, high-grade glioma, survival, fluorescence-guided surgery

## Abstract

Current standard of care for glioblastoma is surgical resection followed by temozolomide chemotherapy and radiation. Recent studies have demonstrated that >95% extent of resection is associated with better outcomes, including prolonged progression-free and overall survival. The diffusely infiltrative pattern of growth in gliomas results in microscopic extension of tumor cells into surrounding brain parenchyma that makes complete resection unattainable. The historical goal of surgical management has therefore been maximal safe resection, traditionally guided by MRI and defined as removal of all contrast-enhancing tumor. Optimization of surgical resection has led to the concept of supramarginal resection, or removal beyond the contrast-enhancing region on MRI. This strategy of extending the cytoreductive goal targets a tumor region thought to be important in the recurrence or progression of disease as well as resistance to systemic and local treatment. This approach must be balanced against the risk of impacting eloquent regions of brain and causing permanent neurologic deficit, an important factor affecting overall survival. Over the years, fluorescent agents such as fluorescein sodium have been explored as a means of more reliably delineating the boundary between tumor core, tumor-infiltrated brain, and surrounding cortex. Here we examine the rationale behind extending resection into the infiltrative tumor margins, review the current literature surrounding the use of fluorescein in supramarginal resection of gliomas, discuss the experience of our own institution in utilizing fluorescein to maximize glioma extent of resection, and assess the clinical implications of this treatment strategy.

## Introduction

Glioblastoma is the most common and aggressive primary brain cancer, with over 12,000 new cases diagnosed each year (1). Current standard of care is surgical resection followed by radiation and temozolomide chemotherapy. Even with maximal therapy, prognosis remains bleak, with a median overall survival of 15 months and a 5-year survival of less than 7% (1–3). Despite focused efforts to develop new systemic treatment strategies led by an increasingly nuanced understanding of the molecular underpinnings of this disease, this work has yet to translate into significant clinical benefit for the majority of patients.

Surgical resection remains the mainstay of treatment. The only modifiable prognostic indicator of outcome in glioblastoma is extent of resection, which has been shown to correlate closely with improved survival. The current standard of surgical treatment is maximal safe resection (4, 5). However, because of their diffusely infiltrative growth pattern, which results in the microscopic extension of tumor cells along white matter tracts and into adjacent brain parenchyma, gliomas cannot be completely resected. Maximal or gross total resection, therefore, is currently defined as the removal of all contrast-enhancing tumor. Nonetheless, based on multiple studies employing modern histology as well as advanced sequencing methods, numerous and phenotypically diverse infiltrating glioma cells have been identified in the non-enhancing edematous regions adjacent to the tumor core. This infiltrating tumor margin is currently an area of intense research as the putative region of therapeutic resistance and glioma recurrence (6–8).

Despite advances in surgical management, all patients with glioblastoma eventually experience disease recurrence. Importantly, this recurrence almost always arises within 2 cm of the original lesion, thought to be related to the residual microscopic disease (9, 10). More recently, there has been growing interest in exploring the clinical benefit of extending the cytoreductive goal beyond the borders of the contrast-enhancing tumor, so-called supramarginal or supramaximal resection, to target this microscopically infiltrated region surrounding visible tumor. Over the past decade, there have been a number of studies investigating supramarginal resection for the management of high-grade gliomas, many of which have demonstrated that this technique is associated with improvements in survival compared to gross total resection (11). However, as with any surgical approach in the brain, the benefit of supramarginal resection must be balanced against the risk of causing new neurologic deficit. Furthermore, decisions on extent of resection of infiltrative margins have largely been arbitrary. Imaging and/or intraoperative fluorophores, in combination with other novel intraoperative methods such Raman spectroscopy, will increase the efficacy and safety of resection at the infiltrative margins (12, 13).

In pursuit of increasing the safety and accuracy of brain tumor resection, various technologies have been developed with the goal of providing more reliable intraoperative guidance. Frameless stereotactic neuronavigation is the method most typically used in operating rooms today but is not without its weaknesses. This technology relies heavily upon accurate registration prior to surgery, and even with initial accuracy, as tumor resection progresses brain shift inevitably develops and compromises the precision of localization. Compounding this issue is the fact that deeper areas of tumor, which are often the more dangerous areas with the greatest need for reliable guidance due to their proximity to white matter tracts and vascular structures, are most affected by this phenomenon. Intraoperative MRI has been explored as an alternative means of providing more up-to-date navigational information but is disruptive to the operating room workflow; it also fails to provide true real-time information and represents a significant capital investment (14). Intraoperative ultrasound is lower cost, widely available, and produces less interruption of the surgical workflow, but the quality of the scan is operator dependent, and unfamiliarity with the technique due to lack of standard ultrasound training results in difficulty interpreting images and an extended learning curve (15).

Fluorescent agents that selectively localize to pathologic tissue and can be viewed intraoperatively, such as 5-aminolevulinic acid (5-ALA), do provide genuine real-time feedback. 5-ALA has been extensively studied in glioma neurosurgery and is proven to significantly increase extent of resection and improve patient survival (16). Fluorescein is another fluorophore routinely used in ophthalmology with increasing popularity in neurosurgery. Compared to 5-ALA, fluorescein offers several advantages. 5-ALA is expensive, patients must avoid exposure to light after administration due to the risk of severe skin reactions, and significant time must elapse between drug administration and initiation of surgery. 5-ALA can also be disruptive to surgical workflow due to darkening of the surgical field surrounding the tumor, which may necessitate frequent switching between filtered and white light in order to adequately visualize vascular structures or non-tumor tissue (17, 18). In contrast, fluorescein is low-cost, causes few adverse effects, and is conveniently given during induction of anesthesia (17). In our experience, illumination of the surgical field using a specialized filter for fluorescein allows for clear detection of the fluorescent signal as well as good visualization of the relevant surgical anatomy, a finding that has been supported by others (9, 18).

Fluorescein has been shown to reliably correlate with areas of contrast-enhancement on MRI (19). Most interestingly, it has been demonstrated that fluorescein is also able to localize to non-enhancing regions of low-grade and high-grade gliomas, outside of what would traditionally be considered the area representing surgical goal (9). Here we discuss the potential role of fluorescein in facilitating safer and more targeted supramarginal resection in high-grade glioma, as well as the clinical implications of this treatment strategy.

## What Is the Clinical Benefit of Pursuing Supramarginal Resection?

### Current Knowledge of the Safety and Feasibility of Supramarginal Resection in Glioblastoma

In its most basic sense, supramarginal resection refers to the removal of tumor exceeding “gross total resection”, or, more specifically, the resection of tissue beyond the contrast-enhancing tumor border (11). However, well-defined standardized criteria for what constitutes supramarginal resection have not been established, and different research groups have used different criteria. In their retrospective series of 32 patients, Glenn et al. defined supramarginal resection as the removal of all contrast-enhancing tissue plus at least 1 cm of surrounding brain (20). Studies by Li et al., Mampre et al., and Pessina et al. all based their supramarginal resection on the removal of additional tissue from the region of FLAIR abnormality, but with differing specifications for what percentage of FLAIR tissue must be removed (21–23). Recently, Certo et al. published a study in which supramarginal resection was guided by FLAIR signal with additional assistance from 5-ALA (24). Perhaps most notably, Roh et al. achieved supramarginal resection by performing either a frontal or temporal lobectomy (25). Still other studies have incorporated the utilization of specific dissection techniques into the definition of supramarginal resection (26).

Despite these differences, almost all clinical studies investigating supramarginal resection for glioblastoma have shown that compared to gross total resection, supramarginal resection correlates with prolonged survival, with reported increases in median overall survival ranging from 5 to 25 months, and increases in progression-free survival as high as 19 months (11, 20, 21, 25). Although these findings are encouraging, it is important to recognize that in addition to the inconsistencies in defining supramarginal resection, much of the existing research investigating this technique comprises small, retrospective studies focusing on a select subset of patients. Before this strategy can find a place in the standard surgical management of glioblastoma, much work remains to be done to further clarify what parameters for supramarginal resection are most clinically useful.

### Supramarginal Resection Extends Cytoreduction Into the Peritumoral Tissue, the Putative Driver of Disease Recurrence

Even with complete resection of the contrast-enhancing tumor, glioblastoma invariably recurs, and recurrence is almost always within centimeters of the original lesion, suggesting that the peritumoral region—the region marked by T2/FLAIR signal and lacking contrast enhancement, hence traditionally left behind following surgical resection—plays an important role in this process (6, 27).

The peritumoral region of diffusely infiltrating gliomas contains many different cell populations, both neoplastic and nonneoplastic, that likely contribute to tumor progression and treatment resistance (28). The clinical importance of neoplastic infiltrating cells occupying this region is reflected in evidence suggesting that these cells differ from the neoplastic cells found in the tumor core. Infiltrating glioma cells express genes that may lead to recurrent disease, such as ECM2 and ANGPT1, which play a role cell migration (7). Studies have demonstrated that tumor cells isolated from the resection margins are more invasive than cells sampled from the tumor core (29–32). Using single cell RNA sequencing, Darmanis et al. found that the molecular signature of infiltrating neoplastic cells obtained from the peritumoral tissue was characterized by increased energy production and inhibition of apoptosis, as well as decreased regulation of cell-cell adhesion (7). Moreover, even across distinct patients, the gene expression signatures of infiltrating neoplastic cells displayed a notable degree of homogeneity, possibly implying a shared mechanism of infiltration (7).

Interactions between neurons and glioma cells *via* neuron-glioma synapses may also contribute to tumor progression (33). Depolarization of the glioma cell membrane is known to increase proliferation, referred to as “activity-regulated glioma growth” (33). Gliomas in turn are thought to increase neuronal excitability, creating a positive feedback loop of tumor growth and progression. It stands to reason that these reciprocal interactions predominantly occur at the tumor margin, which is the region where neurons and glioma cells intermingle, and is also the region targeted by supramarginal resection.

While these different cell populations and interactions within the peritumoral environment represent exciting potential targets for medical therapy, more research is needed to bring these findings into the clinical realm. Currently, no known drug can simultaneously target or disrupt all of the elements in the tumor margin that contribute to glioblastoma progression, and the most effective method of addressing these factors remains surgical resection.

Despite the potential benefits of supramarginal resection in glioblastoma, the question remains: how can this technique be effectively and safely carried out? One of the greatest risks of extending surgical resection beyond the area of contrast-enhancement is the possibility of worsening or causing new neurologic deficit. In addition to extent of resection, patient neurological function is closely associated with outcome. Development of a new neurologic deficit after glioblastoma resection has been correlated with a decrease in overall survival, even if the deficit has resolved by as early as the first post-operative visit (11, 34). For this reason, it is crucial to establish clearer, evidence-based guidelines for defining supramarginal resection, and to supplement any attempt at achieving supramarginal resection with appropriate measures to increase safety, such as intraoperative neurophysiological monitoring, image-based navigational guidance, and fluorophores such as fluorescein. Although focused primarily on low-grade gliomas, a 2019 review by Duffau et al. discusses the merits of a functional-based approach for supramarginal resection of diffuse gliomas, where awake brain mapping using direct electrostimulation is combined with intraoperative neurocognitive monitoring to map cortical-subcortical structures, ultimately reducing the risk of malignant transformation while also preserving patient quality of life (35).

## How Can Fluorescein Facilitate Supramarginal Resection in High-Grade Glioma?

### Fluorescein as a Tool for Achieving Greater Rate of Maximal or “Complete” Resection

Fluorescein sodium is a fluorescent compound with peak excitation in the 460 to 500 nm range and peak emission in the yellow-green part of the spectrum between 540 and 690 nm (36). Though traditionally used in retinal angiography, fluorescein’s role in tumor neurosurgery was explored as early as 1947, with studies by Moore et al. investigating the differential retention of fluorescein in malignant versus benign brain tissue (37, 38). Early studies of fluorescein in neurosurgery used doses as high as 20 mg/kg, which enabled visualization under white light illumination (17, 39, 40). With the development of specialized microscopes bearing integrated fluorescence filters, however, doses as low as 3-5 mg/kg are now frequently used, further increasing the safety and tolerability of this agent and bolstering its appeal as a neurosurgical adjunct (17, 18, 41, 42).

Initially, the ability of fluorescein to identify tumor tissue was used for obtaining diagnostic biopsies, but over time interest has grown in its potential to guide resection in high-grade gliomas. Multiple studies have illustrated the utility of fluorescein in improving the rate of gross total resection, with one trial reporting complete resection in over 80% of patients with high-grade gliomas resected using fluorescein, in contrast to the 30-50% rate historically described in patients undergoing surgical resection with white light only (36, 40, 43–45).

Fluorescein is administered intravenously and works not by accumulating within tumor cells, but rather by extravasating in areas of blood brain barrier damage and collecting in the extracellular space (19, 41). This mechanism of action closely mirrors that of gadolinium, and accordingly, much of fluorescein’s value as a surgical adjunct has thus far been in its ability to serve as an intraoperative equivalent of the radiologic gadolinium signal, essentially enabling the neurosurgeon to visualize the contrast-enhancing region of tumor in real-time without the pitfalls of traditional neuronavigation such as brain shift or errors in registration (19, 46).

### Fluorescein Positivity Extends Beyond the Contrast-Enhancing Region of Tumors and Accurately Predicts Pathologic Tissue

Interestingly, despite the similar mechanism of action shared by fluorescein and gadolinium, studies have pointed to the propensity of fluorescein to localize to areas that fail to enhance on T1-weighted MRI (9, 47, 48). It is not entirely understood why this occurs, although proposed mechanisms include differing degrees of vascular disruption between contrast-enhancing and non-enhancing regions, discrepancies in the vascular permeability of fluorescein and gadolinium, and differences in their patterns of diffusion within the extracellular space (9, 19, 47). Regardless, the same studies have shown that these fluorescein-positive, gadolinium-negative areas do not represent a false positive signal, but in fact frequently identify regions of histologically abnormal tissue. We propose that by staining abnormal tissue beyond what is captured by gadolinium contrast enhancement, fluorescein can extend the surgical resection to include those infiltrated tumor margins that play an important role in disease recurrence.

**Figure 1:**
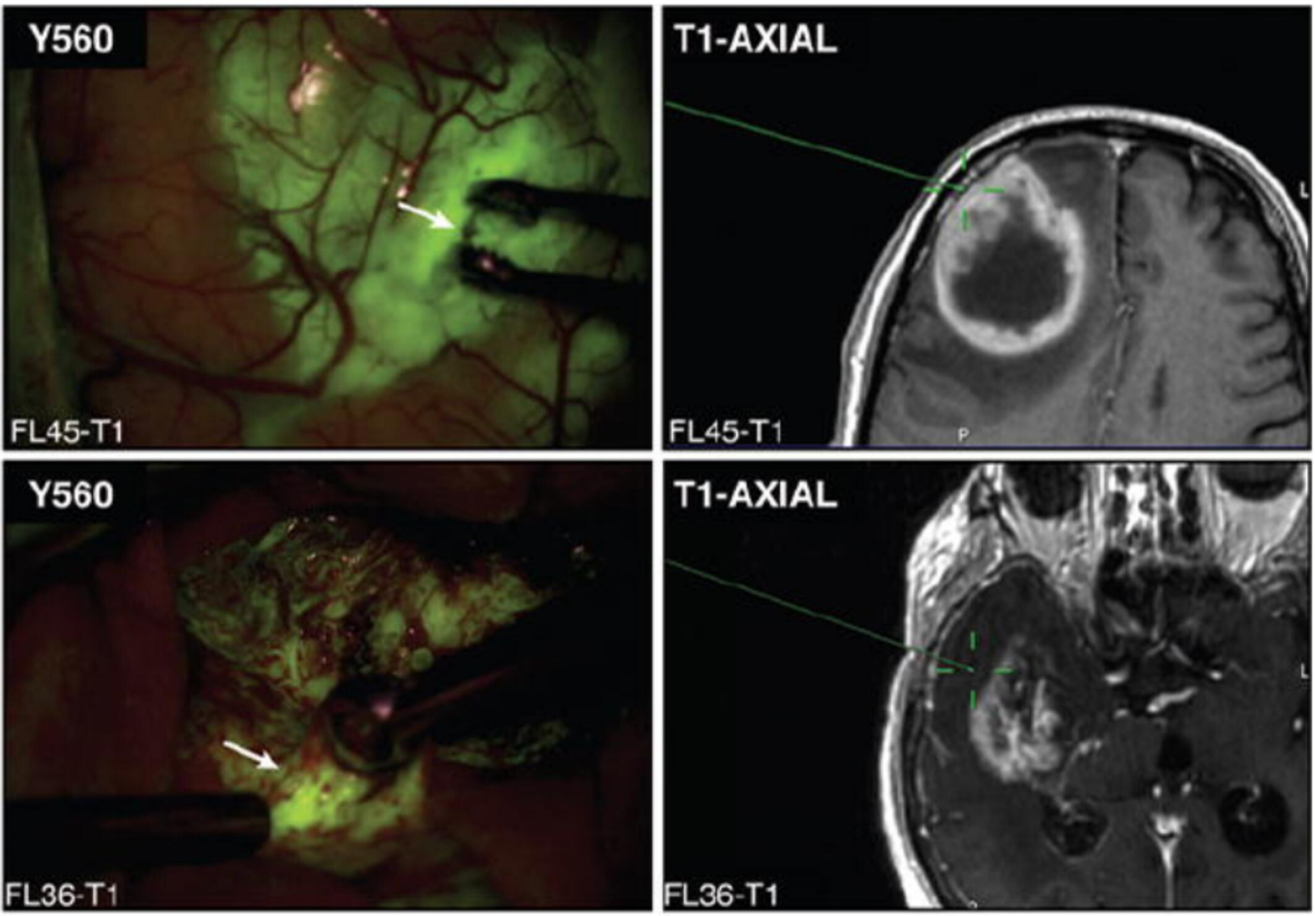
Representative image of biopsy sites (white arrows) from both contrast-enhancing (upper) and non-enhancing (lower) regions, localized using stereotactic guidance with the BrainLab-registered wand indicating the biopsy site on preoperative MRI (green crosshairs). Note that both contrast-enhancing and non-enhancing biopsy sites demonstrate yellow-green fluorescence. Modified from Neira JA: Aggressive resection at the infiltrative margins of glioblastoma facilitated by intraoperative fluorescein guidance. Journal of Neurosurgery 127:111-122, 2017. With permission from Journal of Neurosurgery Publishing Group.

In 2017, our institution published a study exploring the utility of fluorescein for identifying histopathologic alteration in biopsies obtained from both the contrast-enhancing regions and the non-enhancing margins of high-grade gliomas (9). Neira et al. found that fluorescein staining extended beyond the region of contrast-enhancement and into the non-enhancing infiltrative tumor margins (**Figure 1**). Furthermore, samples from these areas had comparable specificity for tumor tissue compared to samples from contrast-enhancing areas. The positive predictive value of subjectively measured fluorescein positivity for histopathologic alteration was >96% in the non-enhancing areas, and 98.6% across all biopsies regardless of radiographic localization, demonstrating fluorescein’s ability to reliably predict glioma-associated pathology in both contrast-enhancing and non-enhancing areas and pointing to its potential role in facilitating more aggressive, supramarginal resection into the non-enhancing margins of high-grade gliomas (9).

Bowden et al. investigated the use of fluorescein in improving diagnostic accuracy of tissue sampling in low-grade gliomas (47). Even in these non-enhancing tumors, fluorescein was able to predict the presence of tissue with pathologic features, once again demonstrating the existence of gadolinium-negative, fluorescein-positive regions that accurately identify pathologic tissue. Although this study sampled only from areas already suspected to be tumor, the authors observed that the fluorescent regions were more likely to demonstrate a greater degree of cytologic atypia, higher cell density, and greater proliferative activity, further supporting our hypothesis that in the absence of contrast enhancement, fluorescein positivity can serve as a marker of areas of interest for surgical resection. In other words, fluorescein can be used to identify regions of high-grade transformation in low-grade gliomas.

In 2018, Schebesch et al. described a series of 5 patients with non-enhancing but positron emission tomography (PET) positive gliomas of varying grade who underwent surgery with fluorescein guidance (48). Specifically, these patients were evaluated with PET using the tracer ^18^F-fluoroethyl tyrosine (FET), which has increased specificity for glioma tissue (49–51). Histopathologic workup of the fluorescent areas in each case was positive for pathologic tissue. Some lesions demonstrated homogenous fluorescence throughout with clear demarcation of the border between diseased and non-affected brain, while others showed more heterogeneous patterns of staining. However, a common theme across all cases was that the distribution of fluorescence closely aligned with that of FET-PET positivity on preoperative imaging, suggesting that there may be some degree of blood brain barrier disruption that traditional gadolinium contrast-enhanced MRI is not sensitive enough to detect, but that can be predicted to an extent based on FET-PET positivity (48). Notably, it has also previously been found that FET-PET can identify non-enhancing, metabolically active tumor (49, 52).

### Limitations of Fluorescein in Achieving Effective Supramarginal Resection

While fluorescein has proven useful in detecting non-contrast-enhancing, glioma-associated tissue, especially at the infiltrative margins, there are limits to this approach. Although fluorescein can certainly contribute to supramarginal resection by identifying regions of interest beyond what is traditionally considered surgical goal, large areas of non-enhancing infiltrated brain that also do not demonstrate fluorescent signal likely remain. In other words, fluorescein does not comprehensively define the boundaries of clinically adequate supramarginal resection. As alluded to earlier, there is still little consensus about what constitutes supramarginal resection, and what definition or criteria best maximizes survival benefit while minimizing patient harm (11). Thus, it will be important to supplement fluorescein with other tools such as intraoperative neurophysiological monitoring (e.g. awake craniotomy, sub-sensory evoked potentials, motor-sensory evoked potentials) and even other fluorescent agents.

## Future Directions

While studies have investigated different means of achieving supramarginal resection in glioblastoma, perhaps the best way to maximize extent of resection is to combine the various navigational tools at our disposal. 5-ALA and fluorescein have distinct mechanisms of action that complement each other in detecting pathologic tissue, and guidance of supramarginal resection using both fluorophores might prove more effective than using either alone (53, 54). Alternatively, fluorescein could be combined with Raman spectroscopy, which has been shown to accurately delineate tumor versus normal brain at the margins of resection, with superior performance compared to 5-ALA (55). Confocal laser endomicroscopy has also been combined with fluorescein and recently investigated as a means of increasing extent of resection by providing real-time intraoperative verification of pathologic tumor tissue (56–58). Several studies have also mentioned the potential benefit of quantitatively measuring fluorescence intensity and integrating this technology with the operative microscope, which could increase both the safety and the sensitivity of fluorescein as an adjunct for glioma resection (9, 17, 29). The utility of fluorescein for resection of recurrent glioblastoma is beyond the scope of this paper, but fluorescein has shown efficacy in identifying recurrent glioblastoma tissue and improving extent of resection in such cases, and might be of use in distinguishing between active disease and pseudoprogression (59). As shown by Bowden et al. and others, fluorescent agents may also serve as promising adjuncts for guiding resection in non-enhancing low-grade gliomas (47, 60). Finally, the development of novel fluorescent probes with even greater specificity for pathologic tissue, for example by labeling brain tissue based on metabolic or molecular alterations, represents an exciting new advancement in the field of fluorescence-guided surgery (61).

## Conclusions

By localizing to areas of pathologic tissue that fail to enhance on T1-weighted MRI, fluorescein may serve as a way of safely and reliably defining targets for supramarginal resection beyond the traditional contrast-enhancing goal of resection. To maximize patient safety, this strategy must be balanced with other well-established tools for increasing the safety and efficacy of surgical resection. Although supramarginal resection for glioblastoma is a promising treatment approach, larger prospective studies are needed to establish a consistent definition of “supramarginal” and determine the extent of resection that maximizes survival benefit while optimizing patient safety. While prolonged survival is an important outcome measure, it is important that these studies also assess the longer-term effects of supramarginal resection on neurological functioning and patient quality of life.

## Author Contributions

LW and MB drafted the manuscript. PC and JB supervised and finalized the manuscript. All authors contributed to the article and approved the submitted version.

## Funding

This research was funded in part through the NIH/NCI Cancer Center Support Grant P30CA013696.

## Conflict of Interest

The authors declare that the research was conducted in the absence of any commercial or financial relationships that could be construed as a potential conflict of interest.
